# Transcatheter Mitral Valve Replacement in Failed Mitral Bioprosthesis: Case Report

**DOI:** 10.1155/cric/2277200

**Published:** 2026-08-02

**Authors:** Veselin Valkov, Nikolay Dragnev, Josef Bis, Nikolay Kalvachev

**Affiliations:** ^1^ First Cardiology Department, UMHAT “St. Marina”, Varna, Bulgaria; ^2^ Department of Propaedeutic of Internal Diseases, Medical University Prof. Dr. Paraskev Stoyanov, Varna, Bulgaria; ^3^ Department of Cardiology and Rheumatology, Medical University Prof. Dr. Paraskev Stoyanov, Varna, Bulgaria; ^4^ Department of Cardiology, Motol and Homolka University Hospital Homolka Campus, Prague, Czech Republic

**Keywords:** degenerated mitral bioprosthesis, iatrogenic atrial septal defect, mitral valve restenosis, neo-LVOT, structural valve degeneration, transcatheter mitral valve replacement, valve-in-valve TMVR

## Abstract

Transcatheter valve‐in‐valve mitral valve replacement (ViV TMVR) is an established therapeutic alternative for selected patients with symptomatic degeneration of a surgical mitral bioprosthesis and high or prohibitive surgical risk. We present a 75‐year‐old woman with a failed Pericarbon More 29‐mm mitral bioprosthesis and severe prosthetic mitral stenosis, which was resolved with transseptal transcatheter ViV TMVR. Baseline echocardiography showed mitral valve area 0.45 cm^2^ by pressure half‐time, mean transmitral gradient 13 mmHg, preserved left ventricular systolic function, estimated systolic pulmonary artery pressure of 55 mmHg, and severe tricuspid regurgitation. ECG‐gated computed tomography was performed for proper procedural planning. During the procedure, after dilation of the interatrial septum, an abrupt left‐to‐right shunt caused hemodynamic compromise. Rapid rescue balloon dilation of the stenotic mitral bioprosthesis and subsequent implantation of a 26‐mm SAPIEN 3 valve restored forward transmitral flow, reduced the interatrial shunt to a minimum, and avoided septal occluder implantation. Postprocedural echocardiography showed a mean transmitral gradient of 3.5 mmHg, no intraprosthetic or paravalvular regurgitation, no LVOT obstruction, and a minimal, hemodynamically insignificant residual shunt. At 6‐month, 12‐month, and 2‐year follow‐ups, the patient remained asymptomatic with stable prosthetic valve function, although the estimated pulmonary artery pressure remained mildly to moderately elevated and tricuspid regurgitation persisted at a moderate degree. Beyond demonstrating the feasibility of valve‐in‐valve TMVR, this case highlights that septal dilation in severe prosthetic mitral stenosis can precipitate abrupt hemodynamic deterioration by creating a clinically significant left‐to‐right interatrial shunt before relief of the mitral obstruction.

## 1. Introduction

Structural valve deterioration (SVD) is an intrinsic and permanent abnormality of a bioprosthetic valve, involving leaflet or supporting‐structure degeneration, calcification, fibrosis, tear, or stent‐frame changes that lead to hemodynamic valve deterioration. In contemporary consensus terminology, SVD should be distinguished from nonstructural valve dysfunction, thrombosis, and endocarditis because the mechanism of bioprosthetic valve dysfunction determines both treatment strategy and follow‐up [1].

In patients with symptomatic prosthetic valve dysfunction, redo surgery remains an important standard treatment option. However, current guideline‐based decision‐making emphasizes heart team assessment, surgical‐risk estimation, anatomic feasibility, and imaging‐based procedural planning. The 2020 ACC/AHA guideline states that a transcatheter valve‐in‐valve procedure is reasonable for high–surgical‐risk patients with prosthetic valve dysfunction at a comprehensive valve center [2], whereas the ESC/EACTS guidelines emphasize heart team care, heart valve centers, and advanced imaging for complex valvular interventions [3].

Valve‐in‐valve TMVR is therefore no longer a novel procedure. The specific clinical message of this case is the anticipation and management of acute hemodynamic deterioration caused by a large iatrogenic left‐to‐right interatrial shunt after septal dilation in the setting of severe prosthetic mitral stenosis. The case illustrates that, before the mitral obstruction is relieved [4, 5], a newly created interatrial communication may become a low‐resistance pathway for left atrial decompression, reducing left ventricular preload and increasing right‐sided volume load. The report also demonstrates how preprocedural CT simulation, rapid restoration of transmitral flow, and postimplant reassessment can guide the decision between septal occluder implantation and conservative management.

## 2. Clinical Case Description

A 75‐year‐old woman was admitted with progressive fatigue, chest discomfort, palpitations, paroxysmal nocturnal dyspnea, and peripheral edema that had developed over 1 month. Five years earlier, she had undergone a surgical aortic and mitral valve replacement with bioprosthetic valves for severe rheumatic aortic and mitral stenosis. Her medical history included permanent atrial fibrillation. Chronic therapy included a loop diuretic, beta‐blocker, calcium‐channel blocker, and acenocoumarol with therapeutic INR control.

On admission, she was hemodynamically stable. Cardiac auscultation revealed an apical diastolic murmur, and pulmonary congestion was present at the lung bases. The electrocardiogram showed atrial fibrillation with a ventricular rate of approximately 80 bpm and a normal electrical axis (Figure 1). Transthoracic and transesophageal echocardiography demonstrated preserved left ventricular systolic function, left atrial enlargement (d = 50 mm, V = 100 mL), normal function of the aortic bioprosthesis, and severe restenosis of the mitral bioprosthesis with thickened, calcified leaflets with reduced mobility (Figure 2). Quantitative echocardiographic findings are summarized in Table 1. The baseline estimated systolic pulmonary artery pressure was 55 mmHg; coronary angiography excluded significant coronary artery disease.

**Figure 1:**
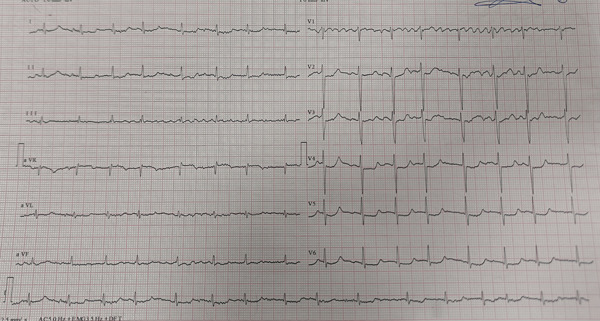
ECG on admission showing atrial fibrillation with an approximate ventricular rate of 80 bpm and normal electrical axis.

**Figure 2:**
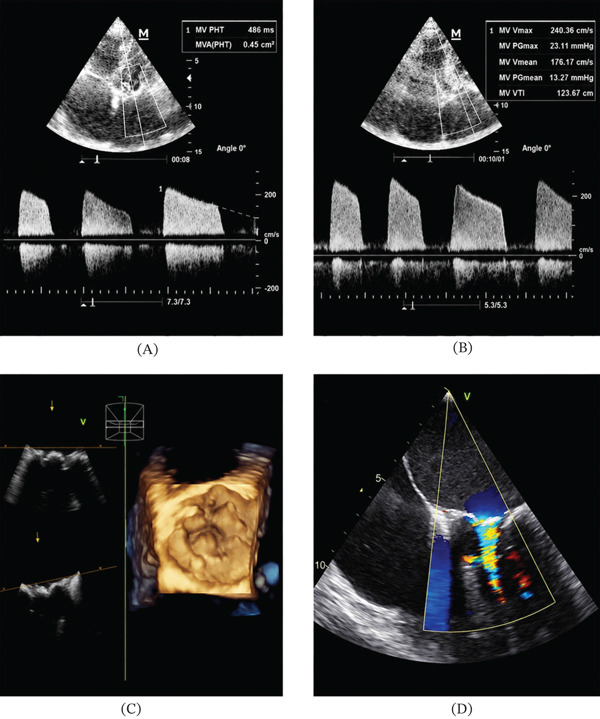
Initial echocardiographic assessment of the degenerated mitral bioprosthesis. (A) PW Doppler trace across the stenotic mitral bioprosthesis showing pressure half‐time of 486 ms and estimated mitral valve area by PHT of 0.45 cm^2^. (B) PW Doppler assessment showing elevated transmitral velocities and gradients across the degenerated bioprosthesis, with peak velocity 2.40 m/s, peak transmitral gradient 23.11 mmHg, mean transmitral gradient 13.27 mmHg, and mitral VTI 123.67 cm. (C) Three‐dimensional transesophageal echocardiography demonstrating severely degenerated and calcified bioprosthetic mitral leaflets with markedly restricted mobility. (D) Color Doppler transesophageal echocardiography showing flow acceleration and inflow convergence across the stenotic mitral bioprosthesis, consistent with severe prosthetic mitral stenosis.

**Table 1:** Serial echocardiographic and hemodynamic parameters before TMVR, after TMVR, and during follow‐up.

Parameter	Pre‐TMVR	Post‐TMVR	Follow‐up 6 months	Follow‐up 12 months	Follow‐up 2 years
Mean transmitral gradient, mmHg	13	3.5	3.81	3.09	4.08
Heart rate during gradient measurement, bpm	~80	~111	~132	~102	~114
Mitral valve area by PHT, cm^2^	0.45	1.4	1.5	1.3	1.4
Indexed mitral valve area, cm^2^/m^2^	0.26	0.82	0.87	0.77	0.82
Left ventricular ejection fraction, %	56	52	49	53	50
TAPSE	15	16	19	17	16
Tricuspid regurgitation severity	Severe	Moderate	Moderate	Moderate	Moderate
Estimated SPAP, mmHg	55	40	45	45	50
Residual interatrial shunt	None before puncture	Significant L‐R shunt after septal dilation; minimal after TMVR	Minimal residual L‐to‐R shunt, unchanged	Minimal residual L‐to‐R shunt, unchanged	Minimal residual L‐to‐R shunt, unchanged
LVOT obstruction/LVOT gradient	Not present; CT predicted neo‐LVOT 287.6 mm^2^	PGmax LVOT 13.0 mmHg	No significant LVOT gradient	No significant LVOT gradient	No significant LVOT gradient

*Note:* Pulmonary artery systolic pressure was estimated echocardiographically; right heart catheterization was not performed. Indexed mitral valve area was calculated using body surface area of approximately 1.71 m^2^.

Abbreviations: LVOT, left ventricular outflow tract; PHT, pressure half‐time; SPAP, pulmonary artery systolic pressure; TAPSE, tricuspid annular plane systolic excursion; TMVR, transcatheter mitral valve replacement.

Given the patient′s high‐risk profile, including advanced age, prior double‐valve surgery, permanent atrial fibrillation, and severely elevated, echocardiographically estimated pulmonary artery systolic pressure, the case was discussed by the heart team, and a transcatheter strategy was selected. The degenerated surgical mitral bioprosthesis was a Pericarbon More 29‐mm valve (Sorin Group, Italy). Procedural planning was based on the surgical valve type and true internal diameter, the valve‐in‐valve mitral application [6, 7], echocardiography, and ECG‐gated cardiac CT [8]. CT simulation for a 26‐mm Edwards SAPIEN 3 valve (Edwards Lifesciences, Irvine, California, United States) showed a mitral annular area of 6.1 cm^2^ and predicted systolic neo‐LVOT area of 287.6 mm^2^ (Figure 3). The predicted neo‐LVOT area was above commonly reported high‐risk thresholds of approximately 170–190 mm^2^, and no preventive LVOT‐modification strategy was considered necessary [9, 10].

**Figure 3:**
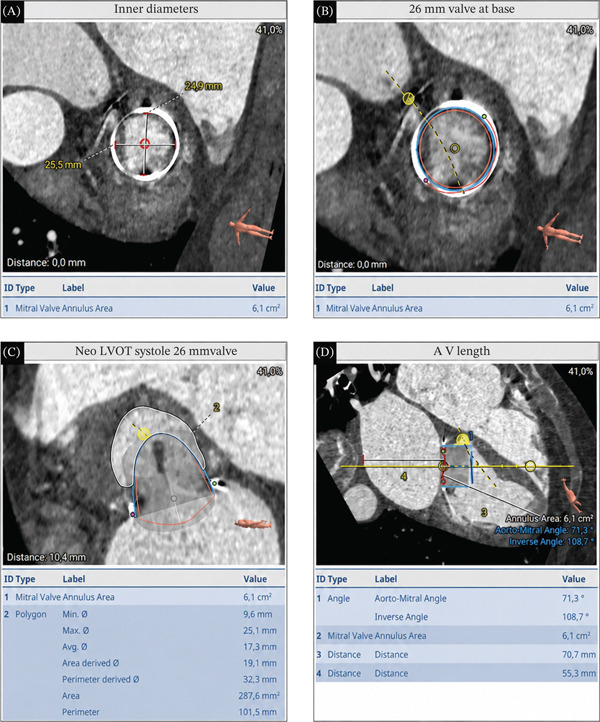
Preprocedural cardiac CT planning. (A) Inner diameters of the degenerated surgical mitral bioprosthesis measured 25.5 and 24.9 mm, with annular area 6.1 cm^2^. (B) Virtual positioning of a 26‐mm transcatheter valve at the base of the surgical bioprosthesis. (C) Systolic neo‐LVOT simulation after virtual implantation of a 26‐mm valve, showing predicted neo‐LVOT area 287.6 mm^2^. (D) CT‐based assessment of aorto‐mitral geometry, showing aorto‐mitral angle 71.3°, inverse angle 108.7°, annular area 6.1 cm^2^, and A–V distances 70.7 and 55.3 mm.

The procedure was performed under general anesthesia with continuous transesophageal echocardiographic and fluoroscopic guidance. A 26‐mm SAPIEN 3 valve was selected for transseptal mitral valve‐in‐valve implantation. After transseptal puncture at the fossa ovalis, the interatrial septum was dilated with a 12/20‐mm Armada balloon (Abbott Vascular, Redwood City, California, United States). Immediately after septal dilation, TEE and hemodynamic monitoring showed a clinically significant left‐to‐right interatrial shunt with rapid hemodynamic deterioration.

The mechanism was interpreted as acute left atrial decompression across a newly enlarged interatrial communication in the setting of severe prosthetic mitral stenosis. Because transmitral forward flow remained critically restricted, the interatrial shunt became a competing low‐resistance exit pathway from the left atrium, reducing left ventricular preload and systemic output while increasing right atrial and right ventricular volume load. Rapid rescue balloon dilation of the stenotic mitral bioprosthesis was therefore performed to restore transmitral flow and stabilize the patient. The SAPIEN 3 valve was then advanced and implanted (Figure 4). Final positioning left approximately 20% of the prosthesis on the atrial side and 80% on the ventricular side, with satisfactory coaxial alignment. After implantation, the interatrial shunt was substantially reduced and remained minimal; therefore, septal occluder implantation was not performed.

**Figure 4:**
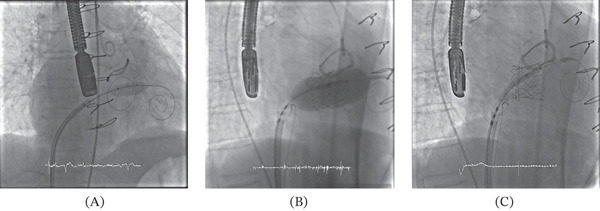
Procedural fluoroscopy. (A) Balloon dilation of the stenotic mitral bioprosthesis after hemodynamic deterioration due to septal dilation. (B) Implantation of 26 mm Edwards SAPIEN 3 valve in the mitral position with slow inflation to allow fine positional adjustment. (C) Final valve position with approximately 80% of the valve in the left ventricle and 20% in the left atrium.

The patient was monitored in the intensive care unit for 24 h. She remained conscious and clinically stable, with no significant clinical and laboratory abnormalities. Predischarge echocardiography showed appropriate valve position and function with mean transmitral gradient of 3.5 mmHg (Figure 5). Intraprosthetic or paravalvular regurgitation, LVOT obstruction, and significant residual interatrial shunt was also excluded. The patient was discharged on the fifth postprocedural day. Therapy at discharge included furosemide 40 mg b.i.d., valsartan 80 mg o.d., bisoprolol 2.5 mg o.d., rosuvastatin 20 mg o.d., and dapagliflozin 10 mg o.d. Anticoagulation with acenocoumarol was continued because of permanent atrial fibrillation and the mitral bioprosthetic position. At 6‐month, 12‐month, and 2‐year clinical and echocardiographic follow‐ups, the patient remained asymptomatic, with normal transcatheter mitral valve function; however, estimated pulmonary artery systolic pressure remained mildly to moderately elevated (45–50 mmHg) and tricuspid regurgitation persisted at a moderate degree, with only limited improvement in right ventricular functional parameters (Table 1; Figure S1).

**Figure 5:**
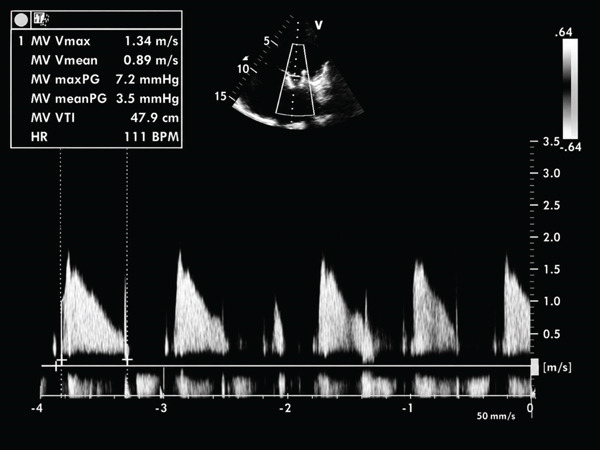
Predischarge echocardiography with pulse‐wave Doppler signal through bioprosthetic valve, showing normal function of the implanted prosthesis with mean transmitral gradient 3.5 mmHg at heart rate 111 bpm.

## 3. Discussion

This case meets contemporary concepts of structural bioprosthetic valve dysfunction because the failed mitral bioprosthesis showed intrinsic leaflet thickening, calcification, and reduced mobility, resulting in severe hemodynamic stenosis. The distinction from thrombosis, endocarditis, paravalvular leak, patient‐prosthesis mismatch, or prosthesis malposition is clinically important because it justified consideration of valve‐in‐valve replacement rather than anticoagulation intensification, infection‐directed treatment, or leak closure [1].

The case aligns with published experience in several ways. The patient was elderly, had prior mitral and aortic valve surgery, and had high surgical risk, which is the typical population in whom transcatheter mitral valve‐in‐valve therapy is considered. Redo mitral valve surgery may be associated with higher perioperative morbidity in elderly and comorbid patients [11, 12], whereas registry and observational data have shown favorable short‐term outcomes for mitral valve‐in‐valve procedures in selected patients [13, 14]. In keeping with guideline‐based principles, the treatment decision depended on heart team assessment, confirmation of surgical valve type and size, and CT‐based anatomic screening.

The unusual and educational feature of the case was the acute deterioration after interatrial septal dilation. Iatrogenic atrial septal defects are expected after transseptal structural interventions, but many are small or clinically silent [4, 5]. In our patient, the hemodynamic context was different: severe prosthetic mitral stenosis generated high left atrial pressure and limited left atrial emptying into the left ventricle. Once the septum had been dilated to permit delivery‐system passage, the new interatrial communication offered a lower resistance pathway for left atrial blood flow into the right atrium. The resulting left‐to‐right shunt likely reduced effective left ventricular preload and stroke volume while acutely increasing right‐sided filling and aggravating the effects of pre‐existing, echocardiographically estimated pulmonary hypertension and tricuspid regurgitation. The immediate treatment priority was therefore rapid relief of the obstructed mitral bioprosthesis to re‐establish forward transmitral flow.

The decision to close or not close an iatrogenic atrial septal defect after TMVR should be individualized [5, 15]. Septal closure should be considered when there is persistent hemodynamic instability after relief of the mitral obstruction, a large residual defect with significant left‐to‐right shunt and right‐sided volume overload, relevant bidirectional or right‐to‐left shunting with hypoxemia, progressive right‐heart failure, or a clinically important calculated shunt fraction such as Qp:Qs in an appropriate clinical context [5, 15]. Conservative management is reasonable when the shunt becomes small or restrictive after valve implantation, systemic hemodynamics are stable, there is no hypoxemia, and follow‐up echocardiography shows no progressive right‐sided dilation or worsening tricuspid regurgitation [15]. In the present case, the shunt decreased to minimal after TMVR, and the patient remained stable; therefore, closure was deferred with planned serial echocardiographic surveillance.

Preprocedural CT was central to procedural safety. TMVR can cause LVOT obstruction because the implanted valve frame and displaced leaflet tissue create a new outflow tract, the neo‐LVOT. Published studies commonly consider predicted neo‐LVOT values below approximately 170–190 mm^2^ to represent increased risk, although risk should be interpreted together with valve type, implantation depth, septal anatomy, left ventricular size, and dynamic systolic geometry [9, 10]. In this patient, the predicted systolic neo‐LVOT area of 287.6 mm^2^ and the absence of immediate postimplant LVOT obstruction supported the original CT‐based risk assessment.

Although valve hemodynamics improved markedly after TMVR and remained stable throughout 2 years of follow‐up, the overall clinical benefit of the procedure should be interpreted with caution. Estimated pulmonary artery systolic pressure, although reduced from the preprocedural value of 55 mmHg, remained elevated at approximately 45–50 mmHg during follow‐up, tricuspid regurgitation persisted at a moderate degree, and TAPSE showed only limited improvement (Table 1). These findings suggest that relief of prosthetic mitral stenosis and elimination of the interatrial shunt did not fully reverse the pre‐existing pulmonary vascular and right ventricular remodeling, which likely reflects a component of longstanding, only partially reversible pulmonary hypertension and right‐sided dysfunction that antedated the intervention. Excellent transcatheter valve performance should therefore not be equated with complete hemodynamic normalization, and patients with long‐standing prosthetic mitral stenosis and secondary pulmonary hypertension may continue to have residual right‐heart limitation despite a successful procedural and valve‐related outcome. It should also be noted that serial transmitral gradients were measured at relatively high heart rates during follow‐up, including approximately 132 bpm at 6 months; because transmitral gradients are heart‐rate dependent, some follow‐up values may modestly overestimate the true resting gradient, which further supports a favorable interpretation of valve performance despite these elevated heart rates.

This case also highlights areas of uncertainty. Although early and midterm mitral valve‐in‐valve outcomes are encouraging [13, 14], long‐term durability in the mitral position remains incompletely defined, especially in elderly patients with multiple prior valve procedures [16]. In addition, antithrombotic therapy after TMVR is not standardized [17]. This patient had a clear independent indication for long‐term anticoagulation because of permanent atrial fibrillation, and acenocoumarol was therefore continued. In patients without atrial fibrillation, the optimal choice and duration of anticoagulation after TMVR remain uncertain and should be individualized according to bleeding risk, valve thrombosis risk, and local heart team practice [17].

## 4. Conclusion

Transseptal mitral valve‐in‐valve TMVR is an important option for selected high‐risk patients with severe structural degeneration of a surgical mitral bioprosthesis. In patients with severe prosthetic mitral stenosis, septal dilation may create a clinically important left‐to‐right shunt before the mitral obstruction is relieved. Prompt recognition and rapid restoration of transmitral flow are crucial for procedural success. Reassessment of residual shunting after valve implantation can guide whether septal closure is necessary. CT‐based neo‐LVOT simulation and serial quantitative echocardiographic follow‐up should be explicitly reported in such cases.

## Funding

No funding was received for this manuscript.

## Consent

The authors declare that patient consent has been obtained for publication of this clinical case. All reasonable steps to maintain confidentiality and anonymization have been taken.

## Conflicts of Interest

The authors declare no conflicts of interest.

## Supporting information


**Supporting Information** Additional supporting information can be found online in the Supporting Information section. Figure S1: Serial echocardiographic follow‐up after transseptal TMVR valve‐in‐valve. At 6 months, Doppler echocardiography demonstrated low transmitral gradients with mean gradient 3.81 mmHg at 132 bpm and PHT‐derived MVA 1.5 cm^2^ (A–B). At 12 months, valve hemodynamics remained stable, with mean gradient 3.09 mmHg at 102 bpm and PHT‐derived MVA 1.3 cm^2^ (C–D). At 2 years, sustained valve performance was observed, with mean gradient 4.08 mmHg at 114 bpm and PHT‐derived MVA 1.4 cm^2^ (E–F).

## Data Availability

The data that support the findings of this study are available from the corresponding author upon reasonable request.
