# Supplementary figures and images for: Transcatheter Mitral Valve Replacement in Failed Mitral Bioprosthesis: Case Report
Source: Case Rep Cardiol. 2026 Aug 2;2026:2277200. doi: 10.1155/cric/2277200 (PMC13430051; doi:10.1155/cric/2277200)

## 6-month follow-up

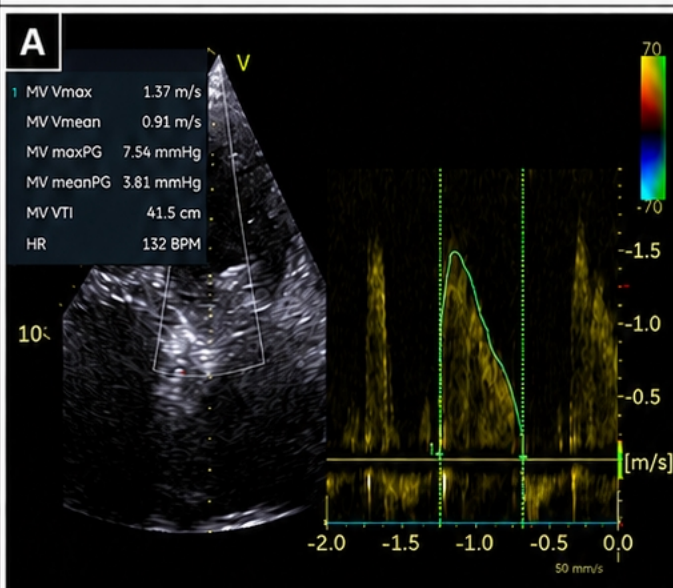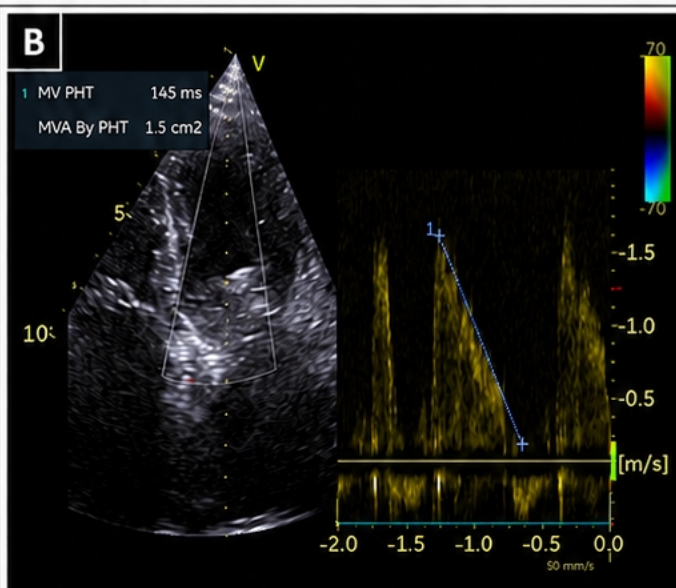

## 12-month follow-up

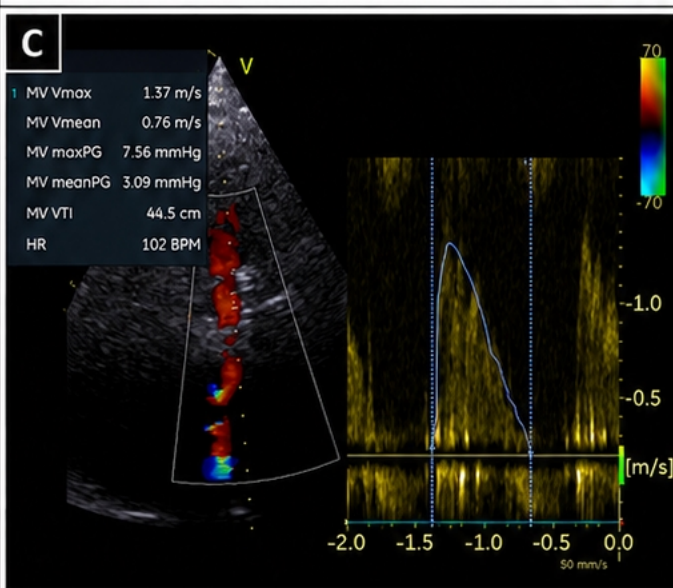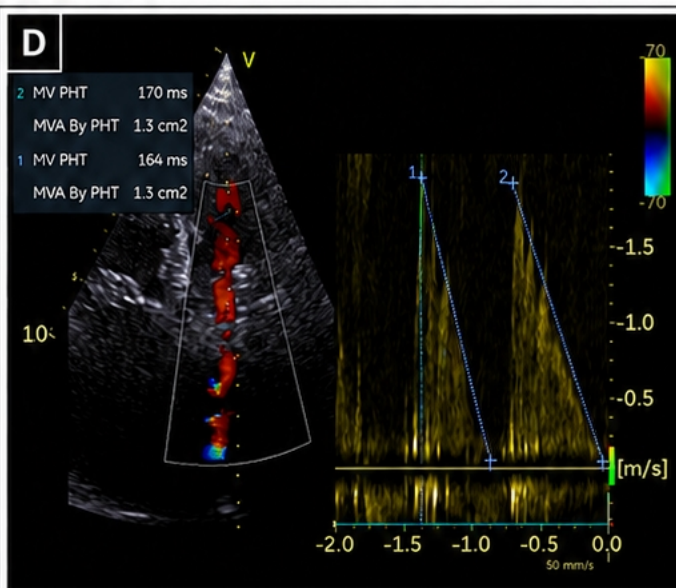

## 2-year follow-up

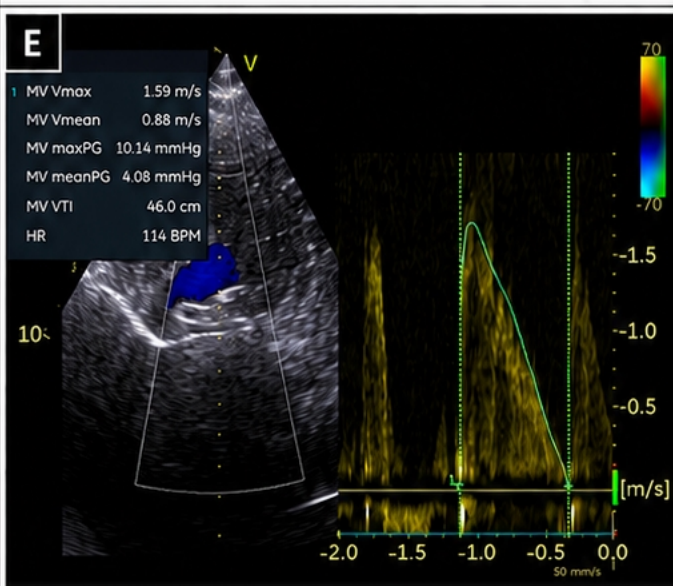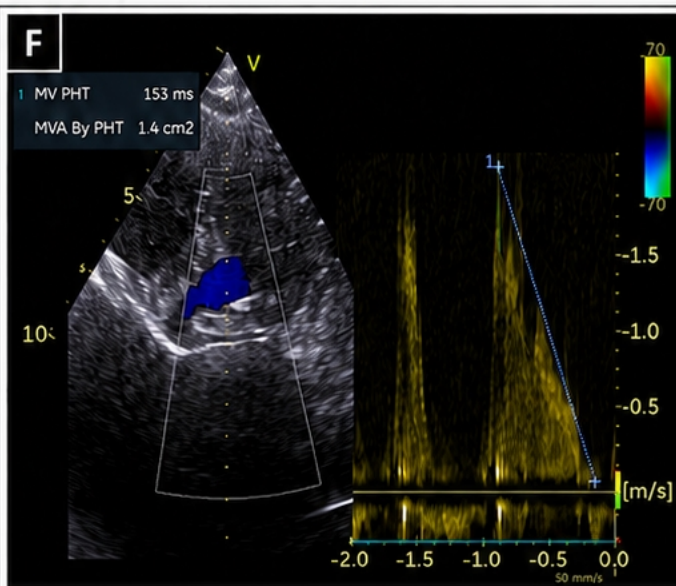

Supplement: Supplementary file 1 — Supporting Information Additional supporting information can be found online in the Supporting Information section. Figure S1: Serial echocardiographic follow‐up after transseptal TMVR valve‐in‐valve. At 6 months, Doppler echocardiography demonstrated low transmitral gradients with mean gradient 3.81 mmHg at 132 bpm and PHT‐derived MVA 1.5 cm2 (A–B). At 12 months, valve hemodynamics remained stable, with mean gradient 3.09 mmHg at 102 bpm and PHT‐derived MVA 1.3 cm2 (C–D). At 2 years, sustained valve performance was observed, with mean gradient 4.08 mmHg at 114 bpm and PHT‐derived MVA 1.4 cm2 (E–F). [file CRIC-2026-2277200-s001.zip › Supplementary Figure S1 UPDATED.pdf]
